# The Impact of Dietary Diversity, Lifestyle, and Blood Lipids on Carotid Atherosclerosis: A Cross-Sectional Study

**DOI:** 10.3390/nu14040815

**Published:** 2022-02-15

**Authors:** Yaqin Wang, Lijun Li, Ying Li, Min Liu, Gang Gan, Yi Zhou, Xiaofei Luo, Chun Zhang, Jianfei Xie, Yinglong Duan, (Andy) S. K. Cheng

**Affiliations:** 1 Health Management Center, The Third Xiangya Hospital of Central South University, Changsha 410013, China; wangy11@cus.edu.cn (Y.W.); ydia0312@csu.edu.cn (Y.L.); 2 Xiangya Nursing School, Central South University, Changsha 410017, China; iamleelj@163.com (L.L.); 18569032268@163.com (G.G.); Yi.zhou@csu.edu.cn (Y.Z.); csuluoxf@163.com (X.L.); zhangchun0211@163.com (C.Z.); 3 Nursing Department, The Third Xiangya Hospital of Central South University, Changsha 410013, China; lium0211@163.com; 4 Department of Rehabilitation Sciences, The Hong Kong Polytechnic University, Hong Kong 999077, China; andy.cheng@polyu.edu.hk

**Keywords:** carotid atherosclerosis, lifestyle, dietary diversity, blood lipids

## Abstract

Carotid atherosclerosis is a common arterial wall lesion that causes narrowing and occlusion of the arteries and is the basis of cardiovascular events. Dietary habits, lifestyle, and lipid metabolism should be considered integrally in the context of carotid atherosclerosis (CAS). However, this area has been investigated less often in China. To understand the prevalence of CAS in China and the impact of dietary diversity and habits, lifestyle, and lipid metabolism on CAS as well as its predictive factors, a cross-sectional study was performed in two northern and southern Chinese tertiary hospitals from 2017 to 2019. Included participants underwent carotid artery color Doppler ultrasonography, blood lipid examination and dietary evaluation. In total, 11,601 CAS patients and 27,041 individuals without carotid artery lesions were included. The prevalence of CAS was 30.0% in this group. High BMI (OR: 1.685, 95% CI [1.315–2.160]), current (1.148 [1.077–1.224]) or ex-smoking (1.349 [1.190–1.529]), abstinence from alcohol ((1.223 [1.026–1.459]), social engagement (1.122 [1.050–1.198]), hypertension (1.828 [1.718–1.945]), and total cholesterol (1.438 [1.298–1.594]) were risk factors for CAS, while higher dietary diversity according to DDS-2 (0.891 [0.805–0.989]), HDL-C (0.558 [0.487–0.639]), sugar-sweetened beverages (0.734 [0.696–0.774]), and no midnight snack consumption (0.846 [0.792–0.903]) were protective factors. This current study demonstrated that higher dietary diversity was a protective factor against CAS in a healthy population. In addition, current recommendations of healthy lifestyle and dietary habits for preventing CAS should be strengthened. In addition, dietary diversity should concentrate on food attributes and dietary balance, rather than increased quantities.

## 1. Introduction

Cardiovascular diseases are the leading cause of mortality and pose a significant socioeconomic burden [1]. In 2015, more than 17 million people died from cardiovascular disease (CVD), accounting for 31% of all global deaths. Globally, 75% of CVD deaths occur in low- and middle-income countries. In these countries, patients with CVD have limited access to effective and equitable health care, leading to delays in the diagnosis and treatment of CVD and premature death [1,2]. Carotid atherosclerosis (CAS) is a common arterial wall lesion that causes narrowing and occlusion of the arteries and is the basis of cardiovascular events [3].

A healthy lifestyle that includes a prudent diet, regular physical exercise, healthy weight maintenance, moderate alcohol consumption, and not smoking contributes to arterial health [4,5,6]. The Malmö Diet and Cancer Study suggested that a healthy diet and adherence to the recommended fiber intake may reduce the risk of peripheral arterial disease [7]. Dietary diversity score (DDS) is an a priori defined dietary quality evaluation index [8]. Dietary diversity is considered to be a key indicator of high diet quality in different populations [9,10], considered to be an important component of a healthy diet [8]. Diversity in dietary choices increases the potential of consuming different nutrients and phytochemicals needed for optimal health. As a result, the risk of diet-related chronic diseases can be reduced [11,12]. Variety in food attributes and a micronutrient-rich diet contribute more to cardiovascular health than increasing nutrient quantity [12], while no association has been revealed between dietary diversity and carotid artery disease episodes.

The lipid infiltration theory also suggests that the development and progression of CAS is associated with abnormalities in lipid metabolism, mainly the accumulation of cholesterol, especially low-density lipoprotein cholesterol (LDL-C), in endothelial cells [13]. Some of the traditional nonmodifiable risk factors for atherosclerosis are age and male sex. Despite efforts to reduce the burden of CVD by addressing traditional risk factors, such as smoking cessation, blood pressure control and total cholesterol, the worldwide increase in obesity and diabetes has offset these prevention efforts [14]. Therefore, dietary habits, lifestyle, and lipid metabolism should be integrally considered in the context of CAS. However, this has been investigated less often in China.

The purpose of this cross-sectional study was to understand the prevalence of CAS in China and the influence of dietary diversity and habits, lifestyle, and lipid metabolism on CAS combinate of both the genders as well as its predictive factors.

## 2. Method

### 2.1. Study Design and Participants

**Figure 1:**
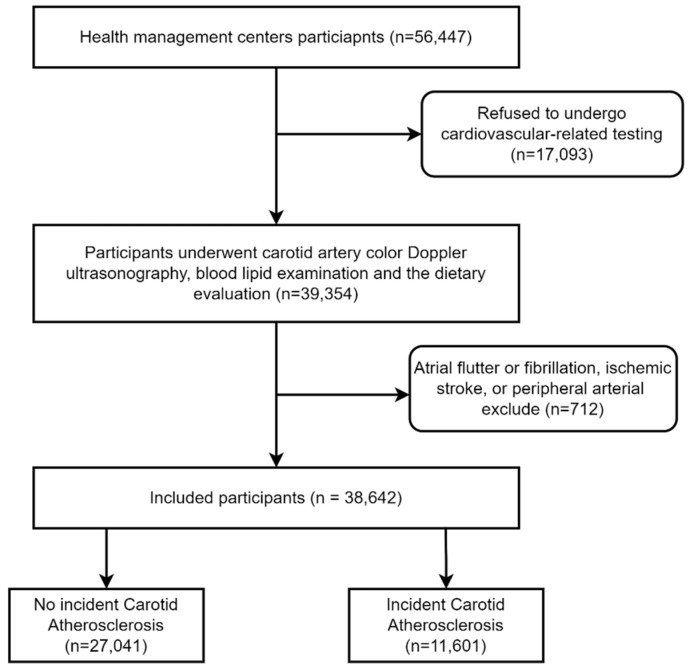
Flowchart of this study.

A cross-sectional study was conducted. The participants were recruited from two health management centers of general tertiary hospitals located in northern and southern China between 1 January 2017 and 31 December 2019. Out of the 17,093 participants excluded, who refused to undergo cardiovascular-related testing, 39,354 participants underwent carotid artery color Doppler ultrasonography, blood lipid examination, and dietary evaluation. This study focused on CAS, so after excluding participants with prevalent atrial flutter or fibrillation, ischemic stroke, or peripheral arterial disease, 38,642 individuals remained enrolled in this study. According to the pathological diagnosis of color ultrasound results, the included participants were divided into patients with carotid atherosclerosis and those without lesions. The two agencies involved in this study provided approval. Participation in the study was entirely voluntary, and there was no reward for participation. Informed consent was obtained from each participant (see Figure 1).

### 2.2. Measures

#### 2.2.1. Individual Characteristics and Lifestyle

The following demographic data were collected from each participant: sex, age, and body mass index (BMI), which was categorized as lean, normal weight, overweight, and obese for BMI < 18.5 kg/m^2^, 18.5–24.9 kg/m^2^, 25–29.9 kg/m^2^, and ≥30 kg/m^2^, respectively. In addition, smoking, alcohol consumption, and exercise were evaluated via a questionnaire. Smoking was defined as never, former, or current smoking or passive smoking. Alcohol consumption was divided into never, current, and abstinent from alcohol. Exercise refers to exercising during leisure time, assessed by whether or not an individual engages in exercise.

#### 2.2.2. Dietary Habits and Dietary Diversity Assessment

Six components of dietary habits were evaluated: (1) the consumption of three meals on time; (2) midnight snacks; (3) overeating; (4) social engagement; (5) sugar-sweetened beverages; and (6) coffee. Each dimension was self-evaluated as “Yes” or “No”.

The Dietary Diversity Scale (DDS) is based on a balanced diet pagoda, which divides foods into nine categories: grains (including cereals, roots, and tubers), vegetables, fruits, livestock meat (including pork, poultry, beef, and organs), fish and shrimp (including seafood, freshwater fish, and aquatic products), eggs, milk, and dairy products, beans (including beans, nuts, and seeds), and oils and fats (including animal and vegetable oil). Food groups other than the abovementioned nine food groups, such as coffee, sugar-sweetened beverages, tobacco, and alcohol, were not included in this score. Each food group is given a score of 1, with a maximum score of 9, independent of the frequency and quantity of the food consumed, based on participants’ recall of the number of food groups consumed in the past three days. If a participant consumed any of the abovementioned foods, they would receive one point in that food category; otherwise, they would receive zero points. Consumption of different foods from the same category will not be double counted. The total score can be up to 9 points. The total score is categorized to three degrees as follows: 1–5 points as insufficient [DDS-1], 6–7 points as moderate [DDS-2], and 8–9 points as sufficient [DDS-3] [15].

#### 2.2.3. Common Risk Factors

Hypertension included a history of hypertension (≥140/90 mmHg) or taking antihypertensive medication. Blood lipid examination results, which included low-density lipoprotein cholesterol (LDL-C), high-density lipoprotein cholesterol (HDL-C), triglycerides, total cholesterol, and blood pressure, were assessed in this study. These parameters have common clinical implications and reflect two different aspects of cardiovascular risk: lipid metabolism and hypertension (blood pressure) [16,17,18].

### 2.3. Statistical Analysis

All collected data were analyzed by SPSS 23.0 for Windows (IBM Corp, Armonk, NY, USA). Descriptive statistics included median (interquartile range (IQR)) and number (percentage) for continuous variables and categorical variables, respectively. Independent samples t tests and χ^2^ tests were used to determine whether participants’ characteristics, dietary habits, and common risk factors were different between patients with and without incident carotid atherosclerosis. To adjust for confounding factors, multilevel logistic regression analysis was used to assess the relationship between dietary habits, lifestyle, common risk factors, and carotid atherosclerosis. Model 1 contained only individual characteristics; Model 2 contained individual characteristics and dietary habits; Model 3 was a full model, adding common risk factors to the variables included in Model 2. Parameters including −2 log likelihood, Nagelkerke R^2^, and omnibus χ^2^ were used to compare these multilevel models. A predefined alpha of 0.05 was used.

## 3. Results

### 3.1. Demographic Characteristics and the Prevalence of CAS

According to the presence or absence of CAS in the participants, the basic individual characteristics, dietary habits, and common risk factors are presented in Table 1. The study included 11,601 CAS patients and 27,041 individuals without carotid artery lesions. The median and IQR of age were 46.0 (37.0–54.0) years old; 59.1% of participants were male, and two-fifths were overweight. Most of the subjects did not smoke (66.8%) or consume alcohol (65.6%) and participated in physical activity (77.6%). Nearly 70% of subjects ate three meals regularly and did not eat midnight snacks or drink coffee. Just 9% reported overeating. Approximately half of the participants consumed sugar-sweetened drinks (44.2%). There were 16.1% participants who reported hypertension. In addition, the median and IQR of HDL-C were 1.30 (1.13–1.52). The prevalence of CAS was 30.0% in the Chinese health examination population.

### 3.2. Bivariate Analysis of Carotid Atherosclerosis

**Figure 2:**
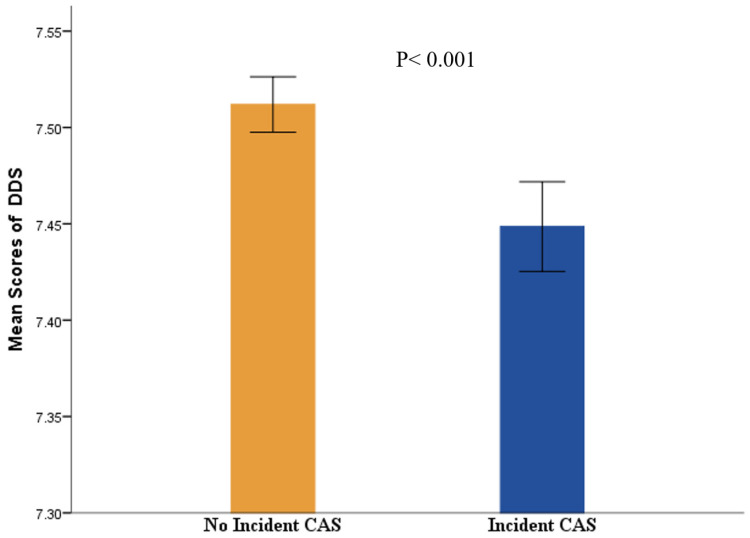
The difference of mean scores of DDS in no incident or incidence of CAS. *Abbreviations:* CAS, Carotid Atherosclerosis; DDS, dietary diversity scale.

As shown in Table 1, the participants with carotid atherosclerosis were significantly older, presented a male predominance, and were more likely to be overweight, current smokers, and alcohol consumers than those without carotid atherosclerosis. According to dietary habits, carotid atherosclerosis participants showed a higher percentage of DDS-1 and a lower percentage of DDS-3 (*p* < 0.001). Participants with CAS had a significantly lower mean DDS score than those without CAS (*p* < 0.001), as presented in Figure 2. Except for eating three meals on time, participants with less carotid atherosclerosis were more likely to eat midnight snacks, overeat, take part in social engagement, and consume sugar-sweetened beverages and coffee than those without carotid atherosclerosis. Regarding the common risk factors for carotid atherosclerosis, participants with carotid atherosclerosis showed a higher percentage (27.0%) of hypertension; the median and IQR of LDL-C [2.99 (2.42–3.54)] mmol/L, triglycerides [1.54 (1.08–2.27)] mmol/L, and total cholesterol [5.19 (4.57–5.85)] mmol/L were higher in individuals with carotid atherosclerosis than in those without carotid atherosclerosis, while HDL-C [1.28 (1.12–1.49)] mmol/L was lower (*p* < 0.001).

### 3.3. Multilevel Logistic Regression Analysis

In the full model (Model 3, Table 2), older subjects (odds ratio (OR) [95% confidence interval (95% CI)]: 1.085 [1.082–1.088]) were more likely to have a risk of carotid atherosclerosis, and females had a lower risk (0.884 [0.833–0.937]). With the increase in BMI, subjects were at progressively higher risk: BMI 18.5–24.9 kg/m^2^ (1.491 [1.193–1.862]), 25–29.9 kg/m^2^ (1.611 [1.286–2.019]), and above or equal to 30 kg/m^2^ (1.685 [1.315–2.160]). Both smokers (1.148 [1.077–1.224]) and ex-smokers (1.349 [1.190–1.529]) exhibited a significantly higher risk. Abstaining from alcohol consumption (1.223 [1.026–1.459]) also increased the risk. Subjects who avoided midnight snacks (0.846 [0.792–0.903]) and liked to consume sugar-sweetened beverages (0.734 [0.696–0.774]) reduced their carotid atherosclerosis risk significantly (*p* < 0.001). Higher degrees of DDS, DDS2 (0.891 [0.805–0.989]), and DDS3 (0.904 [0.818–0.999]) reduced the risk of CAS. Hypertension (1.828 [1.718–1.945]) was a risk factor for carotid atherosclerosis (*p* < 0.001). HDL-C (0.558 [0.487–0.639]) decreased the risk, while total cholesterol (1.438 [1.298–1.594]) increased the risk (*p* < 0.001). Nagelkerke R^2^ = 0.265, −2 log likelihood = 39,233.82, and omnibus χ^2^ = 7988.30 for Model 3.

## 4. Discussion

This current cross-sectional study was conducted in an attempt to understand the association between lifestyle, dietary habits, blood lipids, and carotid atherosclerosis. A higher BMI, total cholesterol, smoking, previous alcohol consumption, and hypertension increased the risk of CAS, while a higher level of DDS, dietary habit of sugar-sweetened beverage consumption, and HDL-C decreased the risk. The overall prevalence of carotid atherosclerosis was 30.0%, which was significantly lower than that in a study of northern China (54.5%) [19], which also included data from the Stroke Screening and Prevention Program.

Increasing attention is being paid to the importance of lifestyle factors in the prevention of atherosclerosis [20]. A higher BMI was significantly associated with the risk of CAS, which was similar to previous studies [21,22]. A cohort study found that obesity is associated with the carotid atherosclerosis index and plaque volume in older adults with type 2 diabetes [22]. Both ex-smokers and current smokers had an increased risk of CAS in our study. Liang et al. also suggested that in both cross-sectional and longitudinal studies, smoking was associated with carotid atherosclerosis in the Chinese middle-aged and elderly population [23]. Although smoking cessation is related to reducing the development of carotid plaque [24], it also plays an important role in the prevention of cardiovascular disease [23]. The difference between these two risk factors may suggest a potential benefit of smoking cessation in reducing the risk of carotid plaque [25]. Ex-smoking was still a risk factor for CAS in this study, even higher than current smoking. A previous study showed that the duration of smoking cessation may have a more significant effect on carotid atherosclerosis in ex-smokers than cumulative smoking exposure [26]. This may be related to the harmful substances in cigarettes that exist even after quitting smoking and that have already caused cardiovascular impairments. Moreover, smoking cessation usually contributes to weight gain. A meta-analysis reported an average weight gain of 4–5 kg after 12 months of quitting, with most weight gain occurring within 3 months of quitting [27]. Weight gain itself increases the risk factors for CAS. An animal experiment suggested that although the metabolic changes of smoking cessation led to weight gain in animals, the accumulation of proatherosclerotic lipids in blood vessels ceased after smoking cessation [28], which showed the smoking cessation still has clinical benefits. Alcohol-abstinent people showed a risk of CAS, while alcohol consumers did not. A previous study suggested that moderate alcohol consumption was associated with a significant protective effect on the development of atherosclerosis in men [29]. Poli et al. reported that “abstainers” may include some former heavy drinkers who may have stopped drinking because of alcohol-related illnesses [30]. Anxiety is a common and recurring symptom in people experiencing alcohol withdrawal, and alcohol-abstinent individuals with anxiety exhibit more severe anxiety [31,32]. Higher plasma cholesterol levels may increase neurosteroid synthesis, which reduces the severity of anxiety and withdrawal symptoms [33]. Therefore, the corresponding psychological programs are needed for abstainers.

DDS-2 and DDS-3 were associated with a decreased risk of CAS. A Belgian cohort study with eight years of follow-up also indicated that dietary diversity scores were significantly associated with femoral atherosclerosis [34]. Previous studies also indicated that DDS is associated with increased consumption of healthy foods, such as vegetables, fruits, and whole grains, rather than meat, and this is positively associated with dietary balance [35]. In contrast, a more varied diet was associated with a significantly higher proportion of total energy from fat and saturated fat and with higher cholesterol intake, especially in poor rural Mexican subjects [36]. This difference may be related to the health philosophy and economic level of the subjects. Higher dietary diversity was negatively correlated to CAS in this study. More diverse food attributes and micronutrient-rich diets, rather than increased quantities of nutrients, contribute to cardiovascular health [12]. It should not be ignored that the participants in this study took the initiative to seek medical examination in the hospital or organized by the units, and this group generally has a fixed income in China. Therefore, the impact of dietary diversity on health should be analyzed specifically for different personal health philosophies and economic conditions, and focus on food attributes and dietary balance.

Sugar-sweetened beverages were also related to a decreased risk of CAS in the current study. Chun et al. suggested that high levels of sugar-sweetened beverage consumption were associated with a higher prevalence and extent of coronary artery calcification [37]. Kurniawan et al. also concluded that sugar-sweetened beverages were associated with an increased risk of dyslipidemia [38]. Individuals who drank sugar-sweetened beverages experienced a better sensory rewarding feel. In addition, Papies expressed that to promote healthy choices, emphasis should be placed on the immediate pleasure gained from drinking healthy beverages rather than its long-term benefits [39]. Perhaps the intake of sugary beverages by the participants in this study did not reach dangerous levels, and people experienced a more sensory and rewarding experience when they drank sweetened beverages than when they drank water, especially if they consume these beverages regularly [39]. Subjects who never ate midnight snacks had a reduced risk of CAS, as opposed to those who took part in social engagement. A previous study showed that frequent late-night snackers and individuals who frequently eat out tend to be obese [40], which is associated with CAS [21].

Hypertension and higher total cholesterol were associated with increased CAS risk in the present study. Hypertension has been associated with carotid atherosclerosis in the general Chinese population [41]. A meta-analysis including 59 studies conducted worldwide also suggested that hypertension is a common risk factor for increased carotid intima-media thickness and carotid plaque in 30- to 79-year-olds [42]. Early detection and management of hypertension may help slow the progression of CAS, and special attention should be given to subpopulations that are particularly vulnerable, such as men and smokers [43]. A high total cholesterol level was a risk factors for CAS in our study, which is similar to the findings of a previous study [44]. Elevated HDL-C levels were found to be an important protective factor, which coincides with a meta-analysis [45] and a cross-sectional study in urban populations in northern China [44]. These situations may be related to the progression of social mechanization and the decrease in physical activity [19].

There were several limitations in this study. First, this was a cross-sectional study, and the results cannot be used to predict the development of CAS. Future prospective cohort studies are indispensable. Second, selection bias needs to be considered. The proportion of health-conscious people in the study population may be higher than that in the general population because the study participants were willing to take the initiative to go to the hospital to participate in the health examination or because of the collective organization of the unit. Moreover, we just evaluated whether the participants were with or without CAS, while the degree of stenosis and plaque composition was not, which is meaningful for the further study to explore the correlation between lifestyle with the degree of stenosis and plaque composition in CAS patients. Lastly, although the participants in this study were from two hospitals in northern and southern China, there is still a need to expand the sample sources to different regions.

## 5. Conclusions

The prevalence of CAS was 30.0% in the northern and southern Chinese health examination populations. Higher BMI, current or ex-smoker status, abstinence from alcohol, social engagement, hypertension, and total cholesterol were risk factors for CAS, while higher DDS degrees, HDL-C, sugar-sweetened beverages, and no midnight snacks were protective factors. The current study demonstrated that higher dietary diversity was a protective factor against CAS in a health examination population and strengthened current recommendations of health lifestyle and dietary habits for preventing CAS. It is worth noting that dietary diversity should concentrate on food attributes and dietary balance, rather than increased quantities.

## Figures and Tables

**Table 1:** Demographic characters of all participants with and without incident carotid atherosclerosis (*N* = 38,642).

Variables	All Participants	No Incident Carotid Atherosclerosis (*n* = 27,041)	Incident Carotid Atherosclerosis (*n* = 11,601)	$t/\;x^{2}$	*p*
1. Individual characteristics					
Age	46.0 (37.0–54.0)	42.0 (34.0–50.0)	53.0 (48.0–58.0)	−85.41	<0.001
Gender				114.62	<0.001
Male	22,819 (59.1)	15,494 (57.3)	7325 (63.1)		
Female	15,823 (40.9)	11,547 (42.7)	4276 (36.0)		
BMI (kg/m^2^)				316.95	<0.001
<18.5	946 (2.4)	821 (3.0)	125 (1.1)		
18.5–24.9	22,187 (57.4)	16,024 (59.3)	6163 (53.1)		
25–29.9	13,651 (35.3)	8937 (33.0)	4714 (40.6)		
≥30	1858 (4.8)	1259 (4.7)	599 (5.2)		
Smoking				190.07	<0.001
Non-smoker	25,819 (66.8)	18,392 (68.0)	7427 (64.0)		
Ex-smoker	1438 (3.7)	6512 (24.1)	3226 (27.8)		
Current	9738 (25.2)	854 (3.2)	584 (5.0)		
Passive-smoker	1647 (4.3)	1283 (4.7)	364 (3.1)		
Alcohol				87.75	<0.001
None	25,344 (65.6)	17,954 (66.4)	7270 (62.7)		
Yes	12,744 (33.0)	8702 (32.2)	4042 (34.8)		
Abstinent from alcohol	674 (1.7)	385 (1.4)	289 (2.5)		
Exercise				17.44	<0.001
None	8670 (22.4)	6224 (23.0)	2446 (21.1)		
Yes	29,971 (77.6)	20,816 (77.0)	9155 (78.9)		
2. Dietary Habits					
DDS Degree				37.71	<0.001
DDS-1	2482 (6.4)	1603 (5.9)	879 (7.6)		
DDS-2	14,517 (37.6)	10,170 (37.6)	4347 (37.5)		
DDS-3	21,643 (56.0)	15,268 (56.5)	6375 (55.0)		
Eating three meals on time				254.15	<0.001
No	12,312 (31.9)	9285 (34.3)	3027 (26.1)		
Yes	26,330 (68.1)	17,756 (65.7)	8574 (73.9)		
Midnight snacks				1247.33	<0.001
No	26,699 (69.1)	17,213 (63.7)	9486 (81.8)		
Yes	11,943 (30.9)	9828 (36.3)	2115 (18.2)		
Overeating				16.86	<0.001
No	35,151 (91.0)	24,492 (90.6)	10,659 (91.9)		
Yes	2491 (9.0)	2549 (9.4)	942 (8.1)		
Social engagement				31.34	<0.001
No	30,382 (78.6)	21,054 (77.9)	9328 (80.4)		
Yes	8260 (21.4)	5987 (22.1)	2273 (19.6)		
Sugar-sweetened beverages				696.38	<0.001
No	21,548 (55.8)	12,898 (51.4)	7650 (65.9)		
Yes	17,094 (44.2)	13,143 (48.6)	3951 (34.1)		
Coffee				135.83	<0.001
No	28,664 (74.2)	19,599 (72.5)	9065 (78.1)		
Yes	9978 (25.8)	7442 (27.5)	2536 (21.9)		
3. Common risk factors (mmol/L)					
BP				1452.96	<0.001
Normal BP	32,406 (83.9)	23,940 (88.5)	8465 (73.0)		
Hypertension	6237 (16.1)	3101 (11.5)	3136 (27.0)		
LDL-C	2.83 (2.31–3.37)	2.76 (2.27–3.29)	2.99 (2.42–3.54)	−21.74	<0.001
HDL-C	1.30 (1.13–1.52)	1.31 (1.13–1.53)	1.28 (1.12–1.49)	5.620	<0.001
Triglycerides	1.39 (0.94–2.13)	1.32 (0.89–2.06)	1.54 (1.08–2.27)	−10.82	<0.001
Total cholesterol	4.97 (4.38–5.62)	4.88 (4.31–5.52)	5.19 (4.57–5.85)	−26.70	<0.001

*p* values were from the *t*-test for continuous variables and from the chi-square test for categorical variables; Continuous variables and categorical variables are presented as the medium (interquartile range (IQR)) and number (percentage); Hypertension was defined as the use of antihypertensive medication or a BP of 140/90 mmHg or higher. Abbreviations: BMI: Body mass index; BP: Blood pressure; DDS, Dietary diversity scale; HDL-C, High-density lipoprotein cholesterol; LDL-C, Low-density lipoprotein cholesterol.

**Table 2:** Multilevel logistic regression analysis of the relationship between carotid atherosclerosis and individual characteristics, diet habits, and common risk factors (*N* = 38,642).

Variables	Odds Ratio [95% Confidence Interval]		
	Model 1	Model 2	Model 3
1. Individual characteristics			
Age	1.093 *** [1.090–1.095]	1.089 *** [1.086–1.092]	1.085 *** [1.082–1.088]
Gender			
Male	1.000	1.000	1.000
Female	0.873 *** [0.825–0.923]	0.867 *** [0.818–0.918]	0.884 *** [0.833–0.938]
BMI (kg/m^2^)			
<18.5	1.000	1.000	1.000
18.5–24.9	1.723 *** [1.383–2.146]	1.708 *** [1.370–2.129]	1.494 *** [1.195–1.866]
25–29.9	2.073 *** [1.661–2.588]	2.049 *** [1.640–2.560]	1.614 *** [1.288–2.023]
≥30	2.362 *** [1.852–3.011]	2.358 *** [1.846–3.010]	1.690 *** [1.318–2.166]
Smoking			
Non-smoker	1.000	1.000	1.000
Ex-smoker	1.165 *** [1.096–1.239]	1.200 *** [1.127–1.277]	1.147 *** [1.076–1.223]
Current	1.361 *** [1.203–1.540]	1.399 *** [1.235–1.584]	1.348 *** [1.189–1.528]
Passive-smoker	0.852 * [0.748–0.971]	0.885 [0.776–1.009]	0.859 * [0.751–0.981]
Alcohol consumption			
None	1.000	1.000	1.000
Yes	1.107 *** [1.047–1.172]	1.090 ** [1.028–1.156]	1.041 [0.981–1.105]
Abstinent from alcohol	1.294 ** [1.087–1.541]	1.279 ** [1.074–1.524]	1.225 * [1.027–1.461]
Exercise			
None	1.000	1.000	1.000
Yes	0.914 ** [0.862–0.969]	0.924 ** [0.870–0.981]	0.949 [0.893–1.008]
2. Dietary Habits			
DDS1		1.000	1.000
DDS2		0.902 * [0.816–0.997]	0.891 * [0.805–0.989]
DDS3		0.912 [0.827–1.007]	0.904 * [0.818–0.999]
Eating three meals on time			
Yes		1.000	1.000
No		1.063 * [1.005–1.124]	1.061 * [1.003–1.123]
Midnight snacks			
Yes		1.000	1.000
No		0.847 *** [0.794–0.903]	0.846 *** [0.793–0.903]
Overeating			
No		1.000	1.000
Yes		1.092 [0.999–1.193]	1.066 [0.974–1.166]
Social engagement			
No		1.000	1.000
Yes		1.134 *** [1.062–1.210]	1.122 ** [1.050–1.198]
Sugar-sweetened beverages			
No		1.000	1.000
Yes		0.070 *** [0.703–0.780]	0.735 *** [0.697–0.774]
Coffee			
No		1.000	1.000
Yes		0.915 ** [0.863–0.971]	0.928 * [0.875–0.986]
3. Common risk factors (mmol/L)			
BP			
Normal BP			1.000
Hypertension			1.828 *** [1.718–1.945]
LDL-C			0.912 [0.821–1.014]
HDL-C			0.557 *** [0.486–0.638]
Triglycerides			0.923 *** [0.886–0.961]
Total cholesterol			1.441 *** [1.300–1.596]
−2 log likelihood	40,271.42	40,042.31	39,233.82
Nagelkerke R^2^	0.233	0.240	0.265
Omnibus χ^2^	6950.70	7179.82	7988.30

Model 1: adjusted for age, gender, smoking, alcohol consumption, and exercise; Model 2: adjusted for age, gender, smoking, alcohol consumption, exercise, DDS, eating three meals on time, midnight snacks, overeating, social engagement, sugary drinks, and coffee; Model 3: adjusted for age, gender, smoking, alcohol consumption, exercise, DDS, eating three meals on time, midnight snacks, overeating, social engagement, sugary drinks, coffee, blood pressure, LDL-C, HDL-C, triglycerides, and total cholesterol. Hypertension was defined as the use of antihypertensive medication or a BP of 140/90 mmHg or higher. Abbreviations: BMI: Body mass index; BP: Blood pressure; DDS, Dietary diversity scale; HDL-C, High-density lipoprotein cholesterol; LDL-C, Low-density lipoprotein cholesterol. * *p* < 0.05; ** *p* < 0.01; *** *p* < 0.001.

## Data Availability

The data presented in this study are available on request from the corresponding author.
